# *Streptococcus suis* Sequence Type 7 Outbreak, Sichuan, China

**DOI:** 10.3201/eid1208.060232

**Published:** 2006-08

**Authors:** Changyun Ye, Xiaoping Zhu, Huaiqi Jing, Huamao Du, Mariela Segura, Han Zheng, Biao Kan, Lili Wang, Xuemei Bai, Yongyun Zhou, Zhigang Cui, Shouying Zhang, Dong Jin, Na Sun, Xia Luo, Ji Zhang, Zhaolong Gong, Xin Wang, Lei Wang, Hui Sun, Zhenjun Li, Qiangzheng Sun, Honglu Liu, Boqing Dong, Changwen Ke, Hui Yuan, Hua Wang, Kecheng Tian, Yu Wang, Marcelo Gottschalk, Jianguo Xu

**Affiliations:** * National Institute for Communicable Disease Control and Prevention, Beijing, People's Republic of China;; † State Key Laboratory of Infectious Diseases Prevention and Control, Beijing, People's Republic of China;; ‡ Sichuan Provincial Center for Disease Control and Prevention, Chengdu, People's Republic of China;; § Université de Montréal, Montreal, Quebec, Canada;; ¶ Guangxi Provincial Center for Disease Control and Prevention, Nanning, People's Republic of China;; # Guangdong Provincial Center for Disease Control and Prevention, Guangzhou, People's Republic of China;; ** Jiangxi Provincial Center for Disease Control and Prevention, Nanchang, People's Republic of China;; †† Jiangsu Provincial Center for Disease Control and Prevention, Nanjing, People's Republic of China;; ‡‡ Guizhou Provincial Center for Disease Control and Prevention, Guiyang, People's Republic of China

**Keywords:** Streptococcus suis, multilocus sequence typing(MLST), pulsed-field gel electrophoresis, meningitis, streptococcal toxic shocklike syndrome (STSS)

## Abstract

An outbreak of *Streptococcus suis* serotype 2 emerged in the summer of 2005 in Sichuan Province, and sporadic infections occurred in 4 additional provinces of China. In total, 99 *S. suis* strains were isolated and analyzed in this study: 88 isolates from human patients and 11 from diseased pigs. We defined 98 of 99 isolates as pulse type I by using pulsed-field gel electrophoresis analysis of *Sma*I-digested chromosomal DNA. Furthermore, multilocus sequence typing classified 97 of 98 members of the pulse type I in the same sequence type (ST), ST-7. Isolates of ST-7 were more toxic to peripheral blood mononuclear cells than ST-1 strains. *S. suis* ST-7, the causative agent, was a single-locus variant of ST-1 with increased virulence. These findings strongly suggest that ST-7 is an emerging, highly virulent *S. suis* clone that caused the largest *S. suis* outbreak ever described.

*Streptococcus suis*, a swine pathogen, is increasing in clinical importance in countries with intensive swine industries (1–4). Infection in humans is considered an occupational disease that affects persons who work in close contact with pigs or pork byproducts (5). Currently, 35 capsular types or serotypes are officially described; *S. suis* serotype 2 is considered the most prevalent and virulent in pigs and humans (6). The first human case was recorded in 1968, and only 200 cases have been subsequently reported globally through June 2005 (5). In some Asian countries, *S. suis* may be the second most common cause of adult streptococcal meningitis (7).

However, a larger outbreak due to *S. suis* serotype 2 emerged in the summer of 2005 in Sichuan Province, China. In total, 215 cases were reported; 38 deaths occurred in 202 villages in 36 counties. New cases were identified in 4 additional provinces in China after the Sichuan outbreak. A striking feature of this outbreak was the unusually high rate of death and streptococcal toxic shocklike syndrome (STSS) as a clinical manifestation. Indeed, reported symptoms included high fever, malaise, nausea, vomiting, and diarrhea, followed by meningitis, subcutaneous hemorrhage, toxic shock, and coma in severe cases. This increased severity of *S. suis* infections in humans, such as the shorter incubation time, more rapid disease progression, and higher death rate, underscores the need to better understand the factors associated with *S. suis* infection.

The aim of this study was to characterize and analyze the causative agent of this unusual outbreak with regard to its virulence and evolution. Using multilocus sequence typing (MLST), we classified the isolated strains into a single sequence type (ST), ST-7 of the ST-1 complex. The ST-1 complex is strongly associated with cases of septicemia and meningitis worldwide (8). *S. suis* ST-7 expressed the proposed virulence markers muramidase-released protein (MRP), extracellular protein factor (EF), and hemolysin (named suilysin) (9–11) and was markedly more cytototoxic to human peripheral blood mononuclear cells (PBMC) than a representative ST-1 strain.

## Methods

### Bacterial Isolation and Identification

All α-hemolytic streptococcal colonies grown on sheep blood agar from specimens obtained from normally sterile sites on humans or diseased pigs were identified as *S. suis* by API-20 STREP system (Biomerieux, WeTech, Beijing, People's Republic of China) (Table). The specific serotype of the isolates was characterized as *S. suis* capsular type 2 by using *S. suis* antisera specific against individual serotypes (provided by the Statens Serum Institut, Copenhagen, Denmark) and confirmed by the coagglutination test, as previously reported (12). PCR assays were used to detect the genes coding for 16S rRNA of *S. suis*, for the capsule of *S. suis* serotype 2 (*cps2J*), and for MRP, suilysin (*sly*), and EF, as reported (9,10,13). PCR results were confirmed by sequencing the synthesized fragments. The expression of the virulence markers MRP, suilysin, and EF was confirmed at the protein level by Western blotting bacterial culture supernatants as previously described (11).

**Table:** *Streptococcus suis* capsular type 2 isolates included in this study*

No. isolates tested	Origin (province)	Host	PFGE profile	MLST profile	Comments
84	Sichuan	Human	Type I	ST-7	Summer 2005, outbreak
1	Jiangxi	Human	Type I	ST-7	Summer 2005, sporadic
1	Guangdong	Human	Type I	ST-7	Summer 2005, sporadic
1	Guangxi	Human	Type I	ST-1	Summer 2005, sporadic
1	Guizhou	Human	Type II	ST-1	Summer 2005, sporadic
1	Jiangsu	Human	Type I	ST-7	Summer 1998, outbreak
8	Sichuan	Pig	Type I	ST-7	Summer 2005, outbreak, diseased pigs
3	Jiangxi	Pig	Type I	ST-7	Summer 2005, sporadic, infected pork
1	Jiangsu	Pig	Type I	ST-7	Summer 1998, outbreak, diseased pig

*PFGE, pulsed-field gel electrophoresis; MLST, multilocus sequence typing.

### Pulsed-field Gel Electrophoresis (PFGE) Analysis

The protocol described by Berthelot-Hérault et al., with modification, was used (14). Cells were restricted with 25 U of *Sma*I (Promega, Sino-American Biotechnology Co, Beijing, People's Republic of China). DNA fragments were resolved by PFGE with 1% SeaKem Gold agarose gels and the CHEF-DR III system (Bio-Rad, Beijing, People's Republic of China). *Salmonella enterica* serovar Braenderup H9812 restricted with *Xba*I was used for molecular weight and size determinations (15). Similarities between restriction endonuclease digestion profiles were analyzed by using BioNumerics software (Applied Maths, Kortrijk, Belgium).

### MLST and Phylogenetic Analysis

Seven housekeeping gene loci described by King et al. for MLST analysis of *S. suis* were used in this study: *cpn*60, *dpr*, *recA*,*aroA*, *thrA*, *gki*, and *mutS* coding for a 60-kDa chaperonin, a putative peroxide resistance protein, a homologous recombination factor, a 5-enolpyruvylshikimate 3-phosphate synthase, an aspartokinase/homoserine dehydrogenase, a glucose kinase, and a DNA mismatch repair enzyme, respectively (8). PCR products were purified by using QIAquick PCR product purification columns (Qiagen, Gene, Beijing, People's Republic of China) and directly sequenced at both ends with an ABI Prism 3700 DNA analyzer system (Perkin Elmer Applied Biosystems, Wellesley, MA, USA) (8). For each isolate, the alleles at each of the 7 loci defined the allelic profile or ST. MLST information in the *S. sui*s database identified the phylogenetic position of isolates collected in this outbreak investigation (8). eBURST was used to identify clonal complexes within *S. suis* and to display the overall structure of the population in the MLST database at http://ssuis.mlst.net (16,17). This database contains 92 previously described STs derived from isolates from various sources, including diseased pigs and human patients from different countries. Bayesian maximum likelihood trees were reconstructed with MrBayes version 3.1.1, and maximum likelihood trees were made with the HKY85 model (18).

### Cytotoxicity Assays

Bacterial cytotoxicity to PBMCs from healthy donors was determined as previously described (19). PBMCs were seeded at a final concentration of 10^6^ cells/mL in 24-well plates and incubated for 4 h at 37°C in the presence of ≈10^7^ CFU/mL *S. suis* strain SC84 and SC22 (selected as 2 representative strains from the Sichuan outbreak and isolated from 2 human patients with STSS) or *S. suis* reference strain 31533 used as a control. Strain 31533, isolated from a pig with meningitis in France, was virulent in pig models of infection and toxic to several types of host cells (19,20). Bacterial cytotoxicity was evaluated by lactate dehydrogenase measurement with Cyto-Tox 96 Cytoxicity Kit (Promega, Madison, WI, USA) according to the manufacturer's instructions. Percentage of cytotoxicity was calculated as (sample optical density [OD]_490_ – OD_0%_)/(OD_100%_ – OD_0%_)×100, where OD_0%_ represents the OD_490_ of noninfected cells and OD_100%_ represents the OD_490_ of cells treated with lysis buffer as indicated by the manufacturer. Empty wells with cell culture medium alone served as blanks.

## Results

In total, 99 *S. suis* strains were isolated in this study (Table): 88 from human patients and 11 from diseased pigs. Of the 88 human isolates, 1 each was from Guangdong, Jiangxi, Guizhou, and Guangxi. From the Sichuan outbreak in the summer of 2005, we found 84 human isolates, distributed in 63 administrative villages of 29 counties, and included them in this study. Two strains, 1 from a human patient and 1 from a diseased pig in Jiangsu during the 1998 *S. suis* outbreak, were also included to determine whether the causative agents for the 2 outbreaks were clonal (21).

Of the 11 pig isolates obtained in 2005, 8 were from Sichuan and 3 were from Jiangxi. One of the 8 isolates from Sichuan, named SC16, originated from 1 diseased pig in a herd. The human patient, who slaughtered and processed the first diseased pig in the same herd, became ill 6 hours later and died 18 hours after the onset of illness. The 3 isolates from Jiangxi were isolated from infected meat samples from a cold storage house. The human patient who processed the meat was infected.

All strains were determined as serotype 2 by using diagnostic antiserum against *S. suis* and confirmed by the coagglutination method with specific type 2 antiserum (12). The identity of the isolates as *S. suis* serotype 2 was further confirmed by positive PCR for the genes coding for the 16S rRNA of *S. suis* and for the capsule of *S. suis* serotype 2 (*cps2J*).

PCR showed all isolates to be positive for the virulence genes coding for MRP, suilysin (*sly*), and EF (9,10). PCR results were confirmed with nucleotide sequencing. Ten selected strains tested positive for suilysin, EF, and MRP by Western blot with specific antibodies, as described previously, which confirmed the expression of these virulence-related genes (11). We saw no indication that *S. suis* was transmitted between humans, and almost all patients had contact with pigs or infected meat (22).

**Figure 1:**
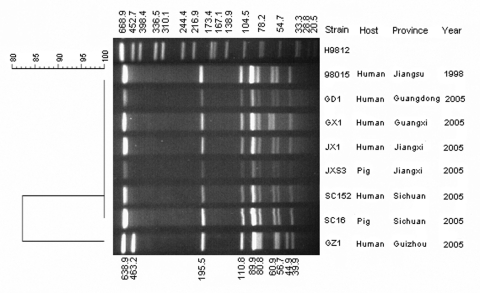
Pulsed-field gel electrophoresis (PFGE) profiles of selected isolates from various parts of China. All isolates of *Streptococcus suis* were digested with *Sma*I. The molecular size of each restriction fragment was calculated with the Comparative Quantification/Polymorphism analysis feature of the Molecular Analyst Fingerprinting Plus software (Quantity One version 4.0, Bio-Rad, Beijing, People's Republic of China) based on mobility of *S. suis* isolates on the same gel with *Salmonella enterica* serovar Braenderup H9812 digested with *Xba*I, as universal standard of PulseNet International. Clustering of PFGE patterns was performed by an unweighted paired group with arithmetic averaging (UPGMA). The dendrogram of PFGE patterns of isolates tested were drawn with PulseNet software BioNumerics, with a 1.5% position tolerance and 1% optimization.

PFGE effectively detected relationships between genetic background, virulence traits, and epidemiologic implications of many bacterial pathogens. Vela et al. analyzed 302 *S. suis* clinical isolates of various serotypes from various countries and identified 60 different pulse types, of which 50% corresponded to a single pulse type. One pulse type represented 46.3% of the swine isolates that may be related to a higher pathogenic potential or to a wider environmental distribution (23). In this study, chromosomal DNA digested with *Sma*I was analyzed in the 101 isolates described in the Table. Results showed 2 pulse types. Pulse type I was found in 100 of the 101 isolates, including those from human patients and diseased pigs from Sichuan and 4 other provinces: Jiangxi, Guangdong, Guangxi, and Jiangsu (Figure 1). This finding indicates that pulse type I was the primary cause of this human outbreak and that the causative agent was clonal. Only 1 isolate, GZ1, isolated in August 2005 and having a slightly different PFGE profile, was from a human patient in Guizhou Province (Figure 1).

Although the PFGE-based network, PulseNet International, is used worldwide for molecular epidemiology, no database is available for *S. suis* to enable comparison of genetic relatedness among isolates from various origins. MLST defines strains by using sequences at 7 housekeeping loci and has become the method of choice for addressing questions related to epidemiology, population, and evolutionary biology. The MLST network, http://www.mlst.net, allows laboratories to quickly characterize their strains, relate them to those submitted by others, and compare them with the pathogen populations as a whole through the Internet (16,24). The selected 7 housekeeping gene loci from all 101 *S. suis* isolates were amplified and sequenced. DNA sequences from the 7 loci of all isolates were determined (DQ205243-51), and allelic profiles were assigned and submitted to the MLST database for *S. suis* (http://ssuis.mlst.net) (8). In our study, 99 of 101 isolates were identified as ST-7, including all 92 isolates from human patients and diseased pigs collected during the Sichuan outbreak in 2005, as well as the 2 isolates from the Jiangsu outbreak in 1998 (1 from a human patient and 1 from a diseased pig). In addition, the isolates from Jiangxi (1 human and 3 pig isolates) and Guangdong (1 human isolate) obtained during summer 2005 were also assigned to ST-7. One human isolate from a patient in Guizhou (strain GZ1) and 1 human isolate from Guangxi (strain GX1) were identified as ST1 (DQ205251). Strain GZ1 showed a PFGE profile that differed slightly from that of the Sichuan isolates (Figure 1, pulse type II). However, strain GX1 had a pulse type I profile similar to that of the Sichuan isolates. These results indicate that MLST is a more powerful discriminatory and epidemiologic tool.

**Figure 2:**
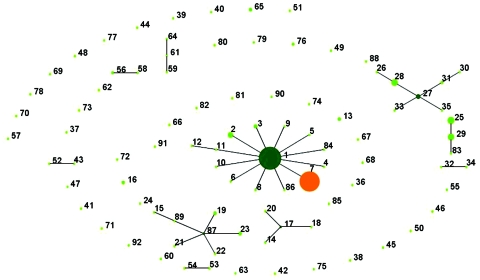
Population snapshot of *Streptococcus suis*. The entire *S. suis* multilocus sequence type database is displayed as a single eBURST diagram. The 6 major sequence type (ST) complexes are each denoted by a number. The patterns of descent within these ST complexes are discussed in the text. Primary founders (dark green) are positioned centrally in the cluster, and subgroup founders are shown in light green, except ST-7, which is shown in orange to emphasize its importance. The area of each circle in the diagram corresponds to the abundance of the isolates of the ST in the input data.

To gain information on the origin and relationships between ST-7 and other STs, we evaluated the population genetic diversity of the entire *S. suis* MSLT database with eBURST, which divides an MLST dataset of any size into groups of related isolates and clonal complexes, predicts the ancestral genotype for each clonal complex, and computes the bootstrap relatedness value. Using this technique, we showed bacterial clonal diversity in *S. suis* and provided evidence for the emergence of new clones of particular clinical relevance (17). The population snapshot of all isolates of *S. suis* generated by eBURST, including our 101 isolates along with all 294 isolates with available MLST data, showed 6 major clusters of related STs and numerous unlinked STs. Of unlinked STs, we noted 6 ST complexes with single-locus variants and some with double-locus variants (8,17). Interspersed among these clonal complexes were minor groups, typically joined doublets and individually unlinked STs that were not single-locus variants of any other STs in the database (Figure 2).

The 6 major clonal complexes with single- and double-locus variants were ST-1, ST-17, ST-27, ST-61, ST-29, and ST-87 (Figure 2). The major clonal complex of *S. suis*, ST-1, contained isolates from humans and pigs that had invasive disease or were healthy carriers and included, besides serotype 2, isolates of serotypes 1, 1/2, 3, 8, 14, and 1/14. ST-1, original strain of the ST-1 complex (with 100% bootstrap support), includes pig isolates from England (83), Spain (44), the Netherlands (3), France (4), and Hong Kong (5); it is the predominant ST responsible for most swine infections worldwide.

ST-7 may be derived from ST-1, the primary strain of the clonal ST-1 complex, and it may have caused the human infections in Sichuan Province. ST-7 (ST profile 1,1,1,1,1,1,3) (DQ205243-50) and ST-1 (ST profile 1,1,1,1,1,1,1) (DQ205243-51) may have diversified by a stepwise accumulation of point mutations. Furthermore, ST-7 may have only very recently diverged, since it showed a high level of similarity in terms of both allelic profiles and sequences to their nonidentical alleles. Thus, the closely related ST-1 and ST-7 share 6 identical loci and have 1 locus, *thyA*, differing only at a single nucleotide (Figure 2). Because the housekeeping genes are recognized as the stable core of the bacterial genome, this divergence further shows the emergence of a new virulent ST.

Using eBURST analysis of the MSLT database, we also examined isolates of *S. suis* serotype 2 from human patients or diseased pigs that have been shown experimentally to be highly virulent in porcine models of infection (8,20,25). Six of the 7 so-called highly virulent strains belonged to ST-1, including 1 strain isolated from a patient with meningitis in England (H11/1) and 1 strain isolated from an abattoir worker with meningitis in Hong Kong (87555). Another highly virulent strain (89-1591) isolated from a pig with septicemia was ST-25, a single-locus variant of the ST-29 complex (26).

## Discussion

**Figure 3:**
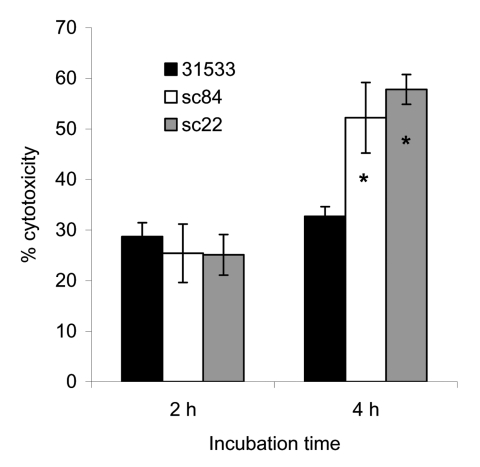
Cytotoxicity of *Streptococcus suis* sequence type (ST) 7 to human peripheral blood mononuclear cells. Data were collected from 3 experiments, which were performed in triplicate and expressed as the mean percentage of cytotoxicity, plus or minus standard deviations. *p<0.001 (compared to the value of *S. suis* ST-1 representative strain 31533) as determined by analysis of variance with SAS version 8 software (SAS Institute, Cary, NC, USA).

Strains isolated from human patients (including those from the MLST database and this study) can be regrouped in either ST-1, ST-6, ST-7, and ST-84 (ST-1 complex) or ST-25 (ST-29 complex) by MLST. ST-1 includes 10 isolates of *S. suis* serotype 2 recovered from human infections in France (n = 4), England (n = 1), and Hong Kong. The Hong Kong strains were isolated in 1985 (n = 1), 1995 (n = 3), and 1997 (n = 1). In addition, ST-1 also includes 1 strain of *S. suis* serotype 14 isolated from a patient in England. ST-25 contains the only 3 serotype 2 strains isolated from human patients in Canada (27,28). ST-6 contains a strain of serotype 14 isolated from a human patient in the Netherlands (29). Before our study, only 1 member of ST-7 had been isolated from the blood of a patient with sepsis in Hong Kong in 1996 (8,30). The ST-1 and ST-29 complexes are highly virulent clones of *S. suis* populations, and the ST-1 clonal complex is the major complex of *S. suis* with public health ramifications and is responsible for most human infections. The 3 isolates in ST-25 have none of the recognized virulence genes in *S. suis*: *ef*, *mrp*, and *sly*. Therefore, we propose that the ST-1 clonal complex is the dominant highly virulent complex and has the potential to cause large human *S. suis* infection outbreaks. The potential of ST-7 as an emerging virulent clone within the ST-1 complex was further suggested by results obtained by using the cytotoxicity assay with human PBMCs. As shown in Figure 3, live bacterial cell suspensions prepared from *S. suis* strains SC84 and SC22 (ST-7), isolated from human patients with STSS in Sichuan, were significantly more toxic to PBMCs than the *S. suis* reference strain 31533 (ST-1) when cells were incubated >2 hours under similar conditions of bacterial growth. Strain 31533 was virulent in mouse and pig models of infection and was toxic to several types of host cells, as demonstrated by in vitro assays (19). Thus, this difference in cytotoxicity is not only significant but also relevant to the fulminant characteristics of the Sichuan outbreak.

**Figure 4:**
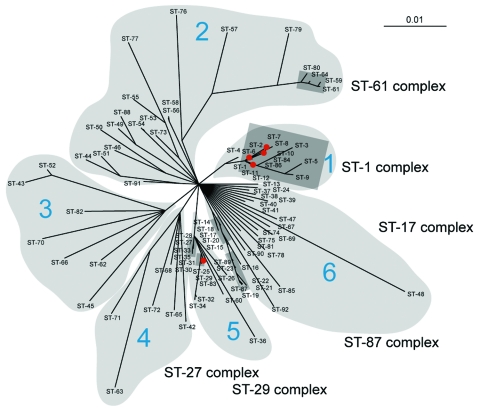
Unrooted Bayesian tree of the concatenated sequence of the 92 sequence types (STs) of *Streptococcus suis*. The tree was constructed by using MrBayes (version 3.1.1) according to the HKY85 model of DNA substitution with no rate variation across sites. Four Markov chains were run for a million generations, and the Markov chain is sampled every 100 generations (18). The sampled parameter values were summarized by discarding the first 2,000 samples as burn-in. On the basis of the last 9,000 samples taken from the posterior probability distribution, a 50% majority rule consensus tree was computed. The posterior probability given on each branch is a percentage of these trees supporting each node. The 6 lineages defined in this study are shadowed in light gray. The 6 major clonal complexes identified previously by King et al. (8) as well as in this study are shadowed in dark gray. The five STs containing isolates from human invasive disease are shown as red dots.

This hypothesis is further supported by phylogenetic analysis of the sequences from 92 STs of the 7 *S. suis* housekeeping genes (Figure 4). To show the phylogenetic relationships between the ST-1 complex and genetic populations of *S. suis*, we examined the possibility that phylogenetic data were sufficient to construct a relatedness tree among members of STs and, in particular, for the virulent clonal ST-1 complex. Phylogenetic analysis showed 6 lineages within the *S. suis* population. The previously identified complexes ST-61, ST-27, and ST-87 were placed in lineages 2, 5, and 6, respectively; most of the STs have only a single isolate. The ST-29 complex that contains human isolates of ST-25 is in lineage 5. The clonal ST-1 complex forms a single lineage and appears to be dominant in various locations (8). It has 4 STs for human isolates, ST-1, ST-6, ST-7, and ST-84, which may have evolved from a common ancestor (Figure 4). In this complex, *S. suis* ST-7 emerged first in Hong Kong in 1996, caused 28 cases in Jiangsu Province in 1998, and was responsible for the largest outbreak of human *S. suis* infection in history that occurred in Sichuan Province, China, in 2005 and resulted in 215 cases with 38 deaths (8,21,30).

The high death rate in the Sichuan outbreak is of great concern since few human *S. suis* infections are fatal. This increased virulence may be related to a horizontal gene transfer of a possible new toxin or superantigen consistent with the most relevant clinical manifestation of this outbreak, i.e., STSS. Recently, resistance gene exchanges were reported between porcine *S. suis* and various human streptococcal species, including *S. pyogenes*. Thus, gene transfer from porcine to human streptococci and vice versa is possible, albeit at a low frequency (31). Therefore, *S. suis* ST-7 might be an emerging virulent infectious disease. This unusual *S. suis* clone may spread further across China and to the rest of the world; spread may become more pronounced with the rapid development of the export business.
